# Quantification of total apolipoprotein E and its isoforms in cerebrospinal fluid from patients with neurodegenerative diseases

**DOI:** 10.1186/s13195-020-00585-7

**Published:** 2020-02-13

**Authors:** K. Minta, G. Brinkmalm, S. Janelidze, S. Sjödin, E. Portelius, E. Stomrud, H. Zetterberg, K. Blennow, O. Hansson, U. Andreasson

**Affiliations:** 1 Department of Psychiatry and Neurochemistry, Institute of Neuroscience and Physiology, the Sahlgrenska Academy at the University of Gothenburg, Mölndal, Sweden; 2 Clinical Neurochemistry Laboratory, Sahlgrenska University Hospital, Mölndal, Sweden; 3 Clinical Memory Research Unit, Department of Clinical Sciences, Lund University, Lund, Sweden; 4 Memory Clinic, Skåne University Hospital, Lund, Sweden; 5 Department of Neurodegenerative Disease, UCL Institute of Neurology, London, UK; 6 UK Dementia Research Institute at UCL, London, UK

**Keywords:** Alzheimer’s disease, Apolipoprotein E, Cerebrospinal fluid, Mass spectrometry

## Abstract

**Background:**

The human *APOE* gene, which codes for apolipoprotein E (apoE), has three major polymorphic alleles: ε2, ε3, and ε4 that give rise to amino acid substitutions. *APOE*-ε4 is a strong risk factor of sporadic Alzheimer’s disease (AD) but the reason why is still unknown despite intense research for more than 20 years. The aim of the study was to investigate if the concentrations of total apoE and the specific apoE isoforms in cerebrospinal fluid (CSF) differ between various neurodegenerative diseases and control individuals, as well as among the *APOE* genotypes.

**Methods:**

Quantification of total apoE and specific apoE isoforms (E2, E3, and E4) in CSF was performed using high-resolution parallel reaction monitoring mass spectrometry. In total, 1820 individuals were involved in the study including clinically diagnosed AD patients (*n* = 228), cognitively unimpaired (CU) patients (*n* = 896), and patients with other neurodegenerative disorders (*n* = 696). Follow-up data was available for 100 individuals, assessed at two time points. Subjects were dichotomized based on an Aβ_42/40_ CSF concentration ratio cut-off into Aβ positive (Aβ+, < 0.091) and Aβ negative (Aβ−, > 0.091) groups.

**Results:**

Even though there was a significant increase of total apoE in the amyloid β-positive (Aβ+) group compared with amyloid β-negative (Aβ−) individuals (*p* < 0.001), the magnitude of the effect was very small (AUC = 0.55). Moreover, CSF total apoE concentrations did not differ between Aβ− CU controls and clinically diagnosed AD patients. There was a difference in concentration between isoforms in heterozygous individuals in an isoform-dependent manner (E2 < E3 < E4) (*p* < 0.001, AUC = 0.64–0.69), and these associations remained when dichotomizing the samples into Aβ+ and Aβ− groups (*p* < 0.01, AUC = 0.63–0.74). In the cohort with follow-up samples, neither total apoE nor isoform-specific apoE concentrations differed between the two time points (*p* > 0.05).

**Conclusions:**

The results indicate that neither the concentrations of total apoE nor the different apoE isoforms in CSF are associated with *APOE-*ε4 carrier status, Aβ status, or clinical dementia diagnoses.

## Introduction

The apolipoprotein E (*APOE*) genotype is closely associated with the risk of Alzheimer’s disease (AD), the most common form of dementia [1]. The human *APOE* gene possesses three major alleles: ε2, ε3, and ε4 with a worldwide frequency of approximately 8%, 78%, and 14%, respectively [2]. *APOE*-ε4 is the strongest genetic risk factor for sporadic AD [3]. Compared to individuals with no *APOE*-ε4, the risk of developing AD is increased two to three times for heterozygous and 12 times for homozygous for the *APOE*-ε4 allele [4] while *APOE*-ε2 is known to be a protective variant against AD [5]. The presence of the *APOE*-ε4 is associated with increased brain atrophy [6], cognitive decline [7], and amyloid deposition [8]. How *APOE*-ε4 contributes to increased risk of AD is, however, still not well understood. The isoform-dependent effects on AD risk might be caused by differential influence of apoE isoforms on the aggregation of amyloid-β (Aβ), which is thought to be a causative agent leading to neurodegeneration in AD [1]. However, it is unclear if *APOE-*ε4 confers a gain of toxic functions (increased Aβ fibrillization), a loss of neuroprotective functions (reduced Aβ clearance), or both [9, 10].

Apolipoprotein E (apoE) is a 299-amino-acid-long (excluding the 18-amino-acid-long signal peptide) glycoprotein [11]. The apoE isoforms differ by single amino acid substitutions with arginine-cysteine interchange at two positions (112 and 158): apoE2 (Cys112, Cys158), apoE3 (Cys112, Arg158), and apoE4 (Arg112, Arg158). These isoforms have different functional and biochemical properties [12].

In the brain, apoE is primarily produced by astrocytes [13], followed by microglia [14] and under pathological conditions apoE can also be synthesized by stressed neurons [15]. ApoE plays an important role in transport of cholesterol and other essential lipids between the cells as a ligand for lipoprotein uptake [9].

AD is preceded by subjective cognitive decline (SCD) and mild cognitive impairment (MCI). ApoE4 is associated with greater memory decline rate and cognitive dysfunction in MCI patients [16], as well as with increased risk of progression from MCI to AD [17]. However, the relationship between apoE and SCD remains unclear [18].

In relation to other types of dementia, the *APOE*-ε4 allele is associated with increased risk of dementia with Lewy bodies (DLB) [19]. On the other hand, most studies have failed to establish any relation between *APOE*-ε4 and susceptibility to Parkinson’s disease (PD) [20, 21].

The possible association between CSF apoE concentrations and AD has been studied extensively with inconclusive outcomes: some studies showed reduced [22, 23], no change [24–27], or increased [28, 29] CSF apoE concentrations in AD patients compared to controls. Regarding other dementias, high CSF apoE concentrations were observed in DLB [30] and PD [31] relative to controls. However, the CSF apoE concentrations in other neurodegenerative diseases, e.g., Parkinson’s disease dementia (PDD), progressive supranuclear palsy (PSP), or multiple system atrophy (MSA), are largely unexplored.

Moreover, isoform-specific apoE concentrations in CSF have not been extensively investigated. Even though no difference was observed in the CSF concentrations of apoE3 and apoE4 isoforms between AD and controls [24], there is no data regarding the apoE2 isoform. The previous study reported an imbalance in apoE isoform concentrations in heterozygotes, where CSF apoE3 levels were higher compared with apoE2 in *APOE-*ε2/ε3 individuals [24]. However, conflicting results were reported regarding the apoE isoform concentrations in *APOE-*ε3/ε4 individuals, where CSF apoE4 levels were either higher compared with apoE3 [26] or equal [24].

Several methods for the quantification of apoE have been introduced. Immunoassays detect total apoE with no certain capability to measure isoforms independently [22, 23, 25, 28, 29], while targeted mass spectrometry (MS) techniques are reliable to discriminate and measure apoE isoforms [24, 26, 27, 32, 33]. Interestingly, the studies using immunoassays for the detection of apoE gave inconclusive results [22, 23, 25, 28, 29], while MS-data was consistent [24, 26, 27, 32, 33].

The aim of the current study was to measure CSF concentrations of total apoE as well as concentrations of specific isoforms in patients with AD and other neurodegenerative diseases in comparison to cognitively unimpaired (CU) Aβ− group to evaluate if there is an association between the concentrations and diagnoses.

## Materials and methods

### Patient samples

All participants gave written informed consent to participate in the study. The study was approved by the regional ethical committee of Lund, Sweden. This project was done as part of the prospective Swedish BioFINDER study (www.biofinder.se).

In total, 1820 individuals with 19 different clinical diagnoses (Additional file 1: Table S1), e.g.*,* healthy controls (*n* = 679), SCD (*n* = 217), MCI (*n* = 309), AD (*n* = 228), PD (*n* = 163), PDD (*n* = 45), and DLB (*n* = 34), were recruited at Skåne University Hospital and the Hospital of Ängelholm, Sweden, between January 2009 and December 2014. The demographics are described in Tables 1, 2, and 3. The inclusion criteria for cognitively healthy elderly were (1) absence of cognitive symptoms as assessed by a physician with special interest in cognitive disorders, (2) age ≥ 60 years, (3) MMSE 28–30 points at screening visit, (4) did not fulfill the criteria for MCI or any dementia disorder, and (5) fluency in Swedish. The exclusion criteria were (1) significant unstable systemic illness or organ failure, such as terminal cancer, that made it difficult to participate in the study, (2) current significant alcohol or substance misuse, and (3) significant neurological or psychiatric illness. The inclusion criteria for patients with SCD or MCI (defined using criteria by Petersen [34]) were (1) referred to a participating memory clinic because of cognitive complaints, (2) age 60 to 80 years, (3) did not fulfill the criteria for any dementia disorder, and (4) fluency in Swedish. The exclusion criteria were (1) significant unstable systemic illness or organ failure, such as terminal cancer, that made it difficult to participate in the study, (2) current significant alcohol or substance misuse, and (3) cognitive impairment that without doubt could be explained by other specific non-neurodegenerative disorders, such as brain tumor or subdural hematoma. Following neuropsychological assessment including a test battery evaluating verbal ability, episodic memory function, visuospatial construction ability, and attention and executive functions, patients were classified as SCD or MCI as previously described [35]. In accordance with the research framework by the National Institute on Aging-Alzheimer’s Association [36], study participants with SCD were analyzed together with the cognitively healthy participants (and combined in the CU group). We also included patients with AD dementia, who fulfilled the DSM-5 criteria for major neurocognitive disorder (dementia) due to AD [36]. The non-AD neurodegenerative diseases group included patients with PDD, DLB, FTD (all fulfilling the DSM-5 criteria for the respective disease), PD (fulfilling the criteria defined by Gelb et al. [37]), PSP (fulfilling the criteria defined by Litvan et al. [38] and Höglinger et al. [39]), and corticobasal syndrome (CBS) (fulfilling the criteria defined by Armstrong et al. [40]).

**Table 1:** Demographics for all patients dichotomized into β-amyloid positive (Aβ+) and β-amyloid negative (Aβ−) groups

Characteristics		Aβ+ (*n* = 778)	Aβ− (*n* = 1039)
Gender, *n* (%)	Male	359 (46%)	512 (49%)
	Female	419 (54%)	527 (51%)
Age, median (IQR)		74 (9)	70 (8)
AD biomarkers, median (IQR)			
MMSE		27 (5)	29 (2)
Aβ_40_, pg/mL		5610 (2703)	5035 (2573)
Aβ_42_, pg/mL		333.2 (182.8)	641.9 (349.7)
Aβ_42/40_		0.061 (0.024)	0.130 (0.029)
t-tau, pg/mL		475.6 (265.9)	274.3 (113.3)
p-tau, pg/mL		82.00 (62.10)	33.74 (15.11)
ApoE, median (IQR)			
Total apoE, μg/mL		3.096 (1.420)	2.964 (1.426)

**Table 2:** Demographics for the sub-cohort dichotomized into β-amyloid positive (Aβ+) and β-amyloid negative (Aβ−) groups

Characteristics		Aβ+ (*n* = 676)	Aβ− (*n* = 755)
Gender, *n* (%)	Male	300 (44%)	338 (45%)
	Female	376 (56%)	417 (55%)
Clinical diagnosis, *n* (%)	AD	202 (30%)	26 (3%)
	MCI	192 (28%)	117 (16%)
	SCD	84 (13%)	133 (18%)
	Controls	198 (29%)	479 (63%)
Age, median (IQR)		73 (8)	71 (8)
AD biomarkers, median (IQR)			
MMSE		27 (5)	29 (2)
Aβ_40_, pg/mL		5851 (2776)	5423 (2474)
Aβ_42_, pg/mL		338.8 (184.0)	697.6 (330.4)
Aβ_42/40_		0.059 (0.024)	0.130 (0.029)
t-tau, pg/mL		496.2 (268.1)	276.5 (110.0)
p-tau, pg/mL		88.16 (64.70)	34.48 (14.56)
ApoE, median (IQR)			
Total apoE, μg/mL		3.112 (1.491)	3.012 (1.465)

The sub-cohort consists of clinically diagnosed AD, MCI, SCD, and controls

**Table 3:** *APOE* genotype frequencies in Aβ+ and Aβ− groups

β-amyloid status	*APOE-*ε2/ε2	*APOE-*ε2/ε3	*APOE-*ε2/ε4	*APOE-*ε3/ε3	*APOE-*ε3/ε4	*APOE-*ε4/ε4
Aβ+ (*n* = 778)	4	20	30	233	376	115
Aβ− (*n* = 1039)	5	143	28	645	208	10

### CSF sampling and analyses of AD biomarkers

Lumbar puncture and CSF handling followed a structured protocol [41]. CSF was analyzed for Aβ42, Aβ40, P-tau181, and T-tau using ELISA (Euroimmun AG, Lübeck, Germany) according to the manufacturer’s recommendations. Samples were dichotomized based on an Aβ_42/40_ CSF concentration ratio cut-off of 0.091 into Aβ positive (Aβ+, < 0.091) and Aβ negative (Aβ−, > 0.091) groups. The cut-off was determined based on the Youden index with [^18^F] flutemetamol PET as the outcome. One PD patient and two controls lacked CSF Aβ_42/40_ values; thus, they were excluded in the analysis regarding Aβ positivity. Follow-up data was available for 100 individuals including two time points separated approximately 2 to 8 years in time (median = 4).

### Selection of peptides

Tryptic peptides unique for isoforms E2, E4, E2/E3, and E3/E4, as well as two peptides common for all three isoforms were selected for monitoring (Additional file 1: Table S2). Corresponding internal standard (IS) peptides purchased from Thermo Fisher Scientific (Ulm, Germany) were labeled with both ^13^C and ^15^N at the C-terminal arginine (Δmass = + 10 Da) of AQUA Ultimate quality (with 98–100% peptide purity and 75–93% peptide content). They were spiked into CSF at equal concentration (0.067 μmol/L) prior to the sample preparation.

### Enzymatic digestion

Sample preparation was performed as described before with minor modifications [42, 43] CSF (20 μL), apoE IS mix (25 μL) were reduced (30 min, 60 °C) with 25 μL of 30 mM dithiothreitol dissolved in ammonium bicarbonate (NH_4_HCO_3_) followed by alkylation (30 min, at room temperature, in dark) with 25 μL of 70 mM iodoacetamide dissolved in NH_4_HCO_3_. Next, the samples were digested (2 h, 37 °C) with 25 μL trypsin/Lys-C mix (Promega Corp., Madison, WI, USA) dissolved in 50 mM NH_4_HCO_3_ to a concentration of 20 μg/mL. Digestion was stopped by adding 25 μL of 10% trifluoroacetic acid. Solid phase extraction (SPE) using Oasis hydrophilic-lipophilic balance (HLB, 2 mg sorbent, 30 μm particle size, Waters Co., Milford, MA, USA) 96-well μElution plates was performed according to the instructions from the manufacturer, with minor modifications: samples were washed with water and eluted using 100% methanol. Samples were then dried in a vacuum centrifuge and stored at − 80 °C pending LC-MS analysis. Three different CSF pools were used as quality controls and were evenly spread out throughout each of the twenty-three 96-well plates used to analyze the study samples.

### Liquid chromatography-mass spectrometry (LC-MS)

Prior to analysis, the samples were reconstituted in 100 μL 50 mM NH_4_HCO_3_. Each sample (50 μL) was loaded onto a Hypersil Gold reversed phase HPLC C18 column (particle size 1.9 μm, id 2.1 mm, length 100 mm, Thermo Fisher Scientific). Mobile phases were A: 0.1% formic acid in H_2_O (v/v) and B: 0.1% formic acid and 84% acetonitrile in H_2_O (v/v/v). Separation was performed at a flow rate of 300 μL/min with a gradient going from 0 to 30% B over 5.5 min using a Vanquish UHPLC (Thermo Fisher Scientific). The gradient was developed to maximize the separation of the peptides (Additional file 2: Figure S1). The total sample cycle time was 10 min. The PRM assay was performed using the Q Exactive hybrid quadrupole-orbitrap high-resolution mass spectrometer (Thermo Fisher Scientific), with electrospray ionization, operated as described previously [43] with some modifications. Briefly, the automatic gain control target value was set to 3 × 10^6^ and maximum injection time to 125 ms. Acquisitions were made at a resolution setting of 35 k. Fragment mass spectra were acquired by scheduled parallel reaction monitoring (PRM) with retention time windows of 30 s for each peptide. Isolation window was set to *m/z* 3 for each peptide, with separate acquisitions of endogenous and IS peptides. The collision energies were optimized manually for each peptide (see Additional file 1: Table S2 for values).

### Data processing

Spectra were acquired using Xcalibur software version 4.1.31.9 (Thermo Fisher Scientific) and imported into Skyline software version 4.1 [44], where fragment ion peak areas were calculated (see Additional file 1: Table S2 and Additional file 3: Figure S2 for selected transitions of precursor and fragment ions and examples of chromatographic traces). Data was then exported from Skyline and further evaluated using in-house developed software. CSF concentrations for each of the apoE peptides were calculated by multiplying the endogenous-to-IS ratios of the summed fragment peak areas by the adjusted concentration of the corresponding IS (Additional file 1: Table S3). Even though the peptide amounts were nominal, the adjustment was needed to improve precision in the quantification. The IS concentrations of the two peptides common for all the three isoforms were adjusted by minimalizing the squared differences of the endogenous concentrations of these peptides (Additional file 1: Table S3). The average of the endogenous concentrations of the two common peptides was used for calculation of total apoE concentration. Next, the IS concentration of the peptide unique for E3/E4 was adjusted by minimalizing the sum of squared differences (Additional file 1: Table S3). The adjustment was performed using the Solver function in Microsoft Excel. Peptides unique for E2/E3 and E3/E4 were used for calculation of isoform-specific apoE concentrations. The isoform quantification was not performed using apoE2 and apoE4 IS concentrations directly due to the high analytical variability of the apoE4 peptide. The quantification of different genotypes was performed by monitoring four isoform-specific peptides: E2, E4, E2/E3, and E3/E4. In the comparisons of apoE isoforms in homozygous individuals (E2/E2, E3/E3, E4/E4), the concentrations of total apoE were divided by two. All six genotypes were identified in this study (Table 3).

### Statistical analyses

The differences between two or more independent groups were investigated using Mann-Whitney *U* test or Kruskal-Wallis test with Dunn’s multiple comparisons, respectively. Area under the curve (AUC) from receiver operating characteristic (ROC) analysis was used as a measure of the effect size. A linear mixed effects model was applied on repeated measures of apoE as dependent variable, time point and covariates (age, gender) as fixed factors and individuals as random factors. Multinomial logistic regression was used to investigate the effect of total apoE or each apoE isoform as covariates on various clinical diagnoses as dependent variable. The analyses were performed using SPSS software, version 25 for Windows (IBM Corp., Armonk, NY, USA) or GraphPad Prism, version 7 for Windows (GraphPad Inc., La Jolla, California, USA). All tests were two-sided and statistical significance was defined as *p* ≤ 0.05.

The CSF cut-off for the Aβ_42/40_ ratio was defined as the intercept between the two normal distributions resulting from an analysis using the *mixtools* package [45] in the statistical software R [46] (Additional file 4: Figure S3).

## Results

The two peptides common for all the three isoforms showed a strong correlation with each other (rho = 0.99) (Additional file 5: Figure S4A), as did their average (total apoE) with the peptides unique for E3/E4 (rho = 0.98) (Additional file 5: Figure S4B) and E2/E3 (rho = 0.98) (Additional file 5: Figure S4C). There is a high correlation between the apoE2 peptide and the difference (total apoE − E3/E4 endogenous peptide) in *APOE-*ε2 carriers (rho = 0.935, *p* < 0.001) (Additional file 6: Figure S5A) and a moderate correlation between apoE4 peptide and the difference (total apoE − E2/E3 endogenous peptide) in *APOE-*ε4 carriers (rho = 0.750, *p* < 0.001) (Additional file 6: Figure S5B).

The analytical coefficient of variations (CVs) of endogenous-to-IS ratios of the four peptides used for apoE quantification in three different CSF pools measured at 12 different occasions were below 13% for both repeatability and intermediate precision. Weighted linear reversed calibration curve fits [47] of the IS-to-endogenous peptide ratios plotted vs eight concentration points of spiked IS (0.002–6.7 μmol/l) in two different CSF pools showed the linearity of the method (Additional file 7: Figure S6). The concentrations of all four measured endogenous peptides used for quantification varied between 0.008 and 0.300 μmol/l, which fits well within the calibration range.

### Total apoE

**Fig. 1:**
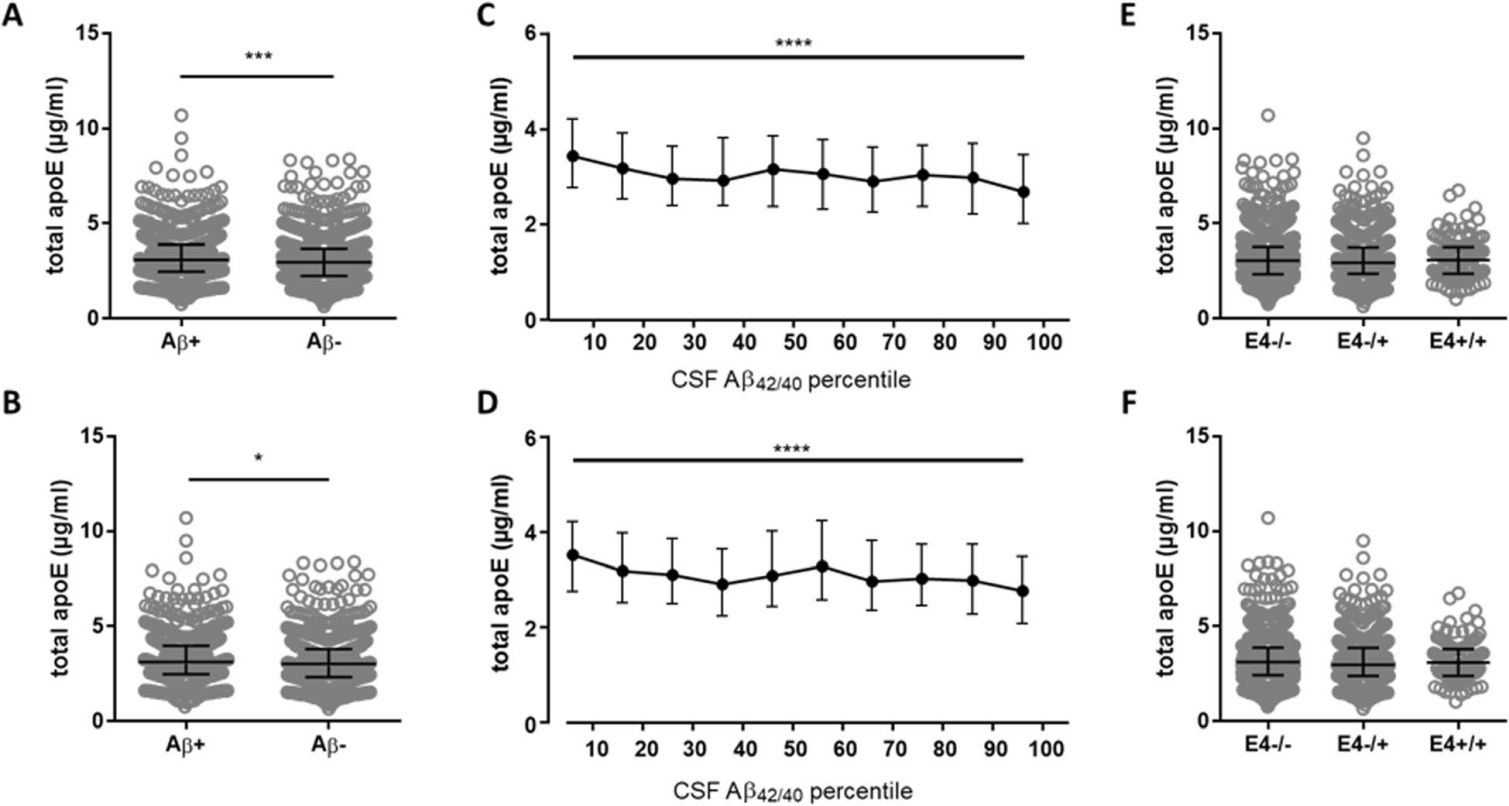
The CSF concentration of total apoE depending on Aβ and *APOE-*ε4 status. The concentration of total apoE for the Aβ+ and Aβ− groups (**a**, **b**), percentile ranges of CSF Aβ_42/40_ (**c**, **d**) and *APOE-*ε4 status (**e**, **f**) for all individuals (**a**, **c**, **e**), and a sub-cohort consisting of AD, MCI, and CU individuals (**b**, **d**, **f**). The horizontal lines represent the median and interquartile ranges. Significance: * = *p* < 0.05, *** = *p* < 0.001, **** = *p* < 0.0001

**Fig. 2:**
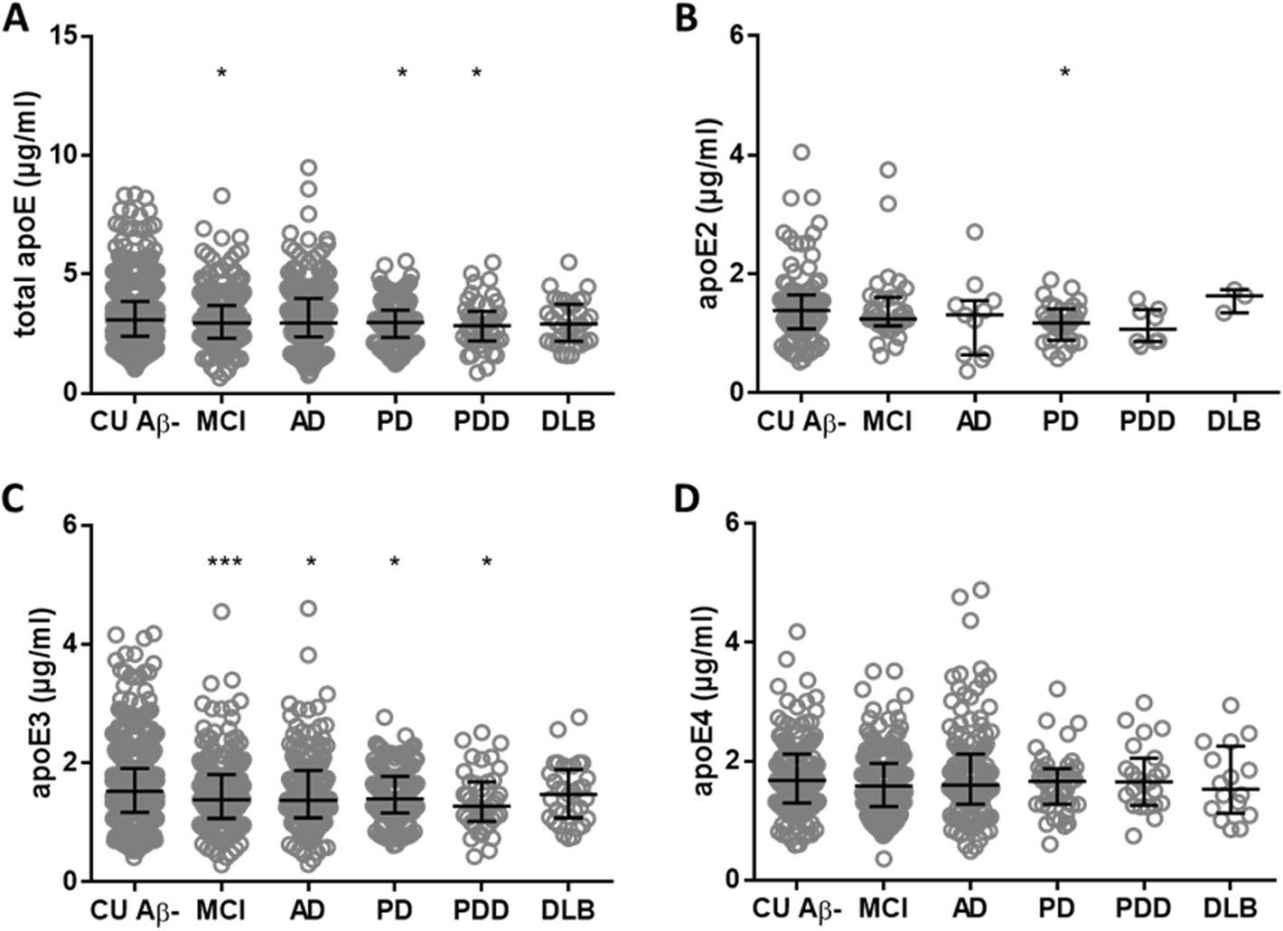
The CSF concentration of apoE in clinically diagnosed neurodegenerative disorders. CSF concentrations of total apoE (**a**), apoE2 (**b**), apoE3 (**c**), and apoE4 (**d**) for cognitively unimpaired Aβ− controls (CU Aβ−), mild cognitive impairment (MCI), Alzheimer’s disease (AD), Parkinson’s disease (PD), pervasive developmental disorder (PDD), and dementia with Lewy bodies (DLB). In the comparisons of apoE isoforms, the individuals were either heterozygous or homozygous for the specific genotype (the concentrations in homozygous individuals were divided by two). The horizontal lines represent the median and interquartile ranges. Significance: * = *p* < 0.05, *** = *p* < 0.001 compared to the CU Aβ− group

There was a significant increase of total apoE in the Aβ+ compared with Aβ− group when taking all the diagnoses together (*n* = 1817, *p* < 0.001) (Fig. 1a), as well as when looking only at the subset consisting of clinically diagnosed AD, MCI, and CU individuals (*n* = 1431, *p* < 0.05) (Fig. 1b). However, the differences were minor (AUC = 0.53–0.55). There was a significant decrease in total apoE with the increase of CSF Aβ_42/40_ from the 0–10 to the 90–100 percentile ranges for all patients (*p* < 0.0001) (Fig. 1c), as well as in the AD-related cohort (*p* < 0.0001) (Fig. 1d). However, the CSF total apoE concentration did not differ between CU Aβ− and clinically diagnosed AD patients (*p* > 0.05, AUC = 0.52) (Fig. 2a). The concentration of CSF total apoE in CU Aβ− group was statistically, but not substantially, higher compared with MCI (*p* < 0.05, AUC = 0.54), PD (*p* < 0.05, AUC = 0.55), and PDD patients (*p* < 0.05, AUC = 0.58) (Fig. 2a). There was no significant difference in CSF total apoE concentration between controls and DLB patients (*p* > 0.05, AUC = 0.54) (Fig. 2a). There was no change in total apoE concentration depending on *APOE-*ε4 status (Fig. 1e, f) and no correlation between total apoE and MMSE was observed (rho = 0.03, *p* > 0.05) (Additional file 8: Figure S7A). There were weak to moderate correlations between total apoE and AD biomarkers for all individuals (rho = − 0.14 for Aβ_42/40_, rho = 0.41 for t-tau, rho = 0.35 for p-tau, *p* < 0.001) (Additional file 8: Figure S7A), for the Aβ+ group separately (rho = − 0.15 for Aβ_42/40_, rho = 0.41 for t-tau, rho = 0.28 for p-tau, *p* < 0.001) (Additional file 8: Figure S7B) and for the Aβ− group (rho = − 0.11 for Aβ_42/40_, rho = 0.52 for t-tau, rho = 0.54 for p-tau, *p* ≤ 0.001) (Additional file 8: Figure S7C).

### ApoE isoforms

CSF apoE2 concentrations were significantly lower in PD patients compared with CU Aβ− group (*p* < 0.05, AUC = 0.63) (Fig. 2b), while no significant difference in apoE2 concentration was observed between CU Aβ− group and other neurodegenerative diseases (Fig. 2b). CSF apoE3 concentrations were significantly higher in CU Aβ− compared with clinically diagnosed MCI, AD, PD, and PDD groups (*p* < 0.05, AUC = 0.55–0.61), but not when compared to DLB patients (Fig. 2c). There was no significant difference in CSF apoE4 concentration between CU Aβ− group and clinically diagnosed MCI, AD, PD, PDD, and DLB (Fig. 2d).

**Fig. 3:**
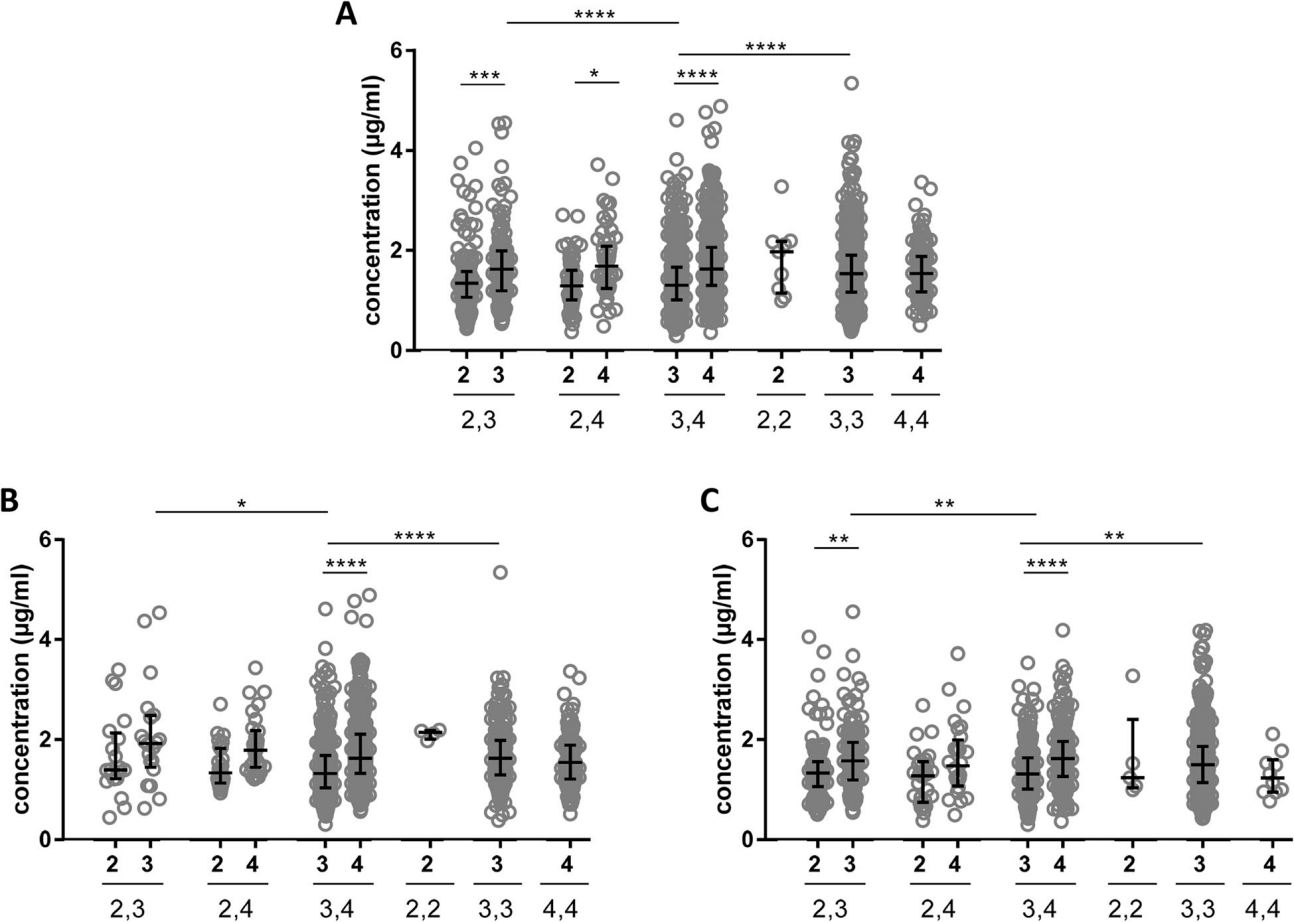
The CSF apoE isoform-specific concentrations. The apoE isoform concentrations are shown for all six genotypes for all patients combined (**a**) and when dichotomized into Aβ+ (**b**) and Aβ− (**c**) groups. In the comparisons of apoE isoforms, the concentrations in homozygous individuals were divided by two. The horizontal lines represent the median and interquartile ranges. Significance: * = *p* < 0.05, ** = *p* < 0.01, *** = *p* < 0.001, **** = *p* < 0.0001

There were significant differences in concentrations between isoforms in heterozygous individuals in an isoform-dependent manner (E2 < E3 < E4) (*p* < 0.05, AUC = 0.64–0.69) (Fig. 3a) and the changes were visible in both groups: Aβ+ and Aβ− (*p* < 0.01, AUC = 0.63–0.74) (Fig. 3b, c). The concentration of apoE4 in *APOE-*ε3/ε4 heterozygous was significantly increased compared with apoE3 in both Aβ+ (*p* < 0.0001, AUC = 0.66) (Fig. 3b) and Aβ− groups (*p* < 0.0001, AUC = 0.66) (Fig. 3c). Additionally, the concentration of apoE3 in *APOE-*ε2/ε3 was elevated in comparison to apoE2 in Aβ− group (*p* < 0.01, AUC = 0.64) (Fig. 3c). When comparing the apoE isoform concentrations between various genotypes, the concentration of apoE3 was decreased in *APOE-*ε3/ε4 patients compared to apoE3,3 and *APOE-*ε2/ε3 patients in all individuals as well as in both Aβ+ and Aβ− groups separately (*p* < 0.05, AUC = 0.53–0.71) (Fig. 3). Unlike apoE3, the concentration of apoE2 and apoE4 did not differ between various genotypes (Fig. 3). ApoE isoform concentrations did not correlate with the MMSE score (rho = 0.02–0.08) (Additional file 8: Figure S7A). There were weak to moderate correlations between apoE3 and apoE4 isoform concentrations and AD biomarkers (Aβ_42/40_, t-tau, p-tau) for all individuals (rho = − 0.14–0.41, *p* < 0.001) (Additional file 8: Figure S7A) as well as for the Aβ+ (rho = − 0.15–0.44, *p* < 0.001) and Aβ− groups (rho = − 0.21–0.59, *p* < 0.01) (Additional file 8: Figure S7B-C). CSF apoE2 concentrations did not correlate with Aβ_42/40_ (Additional file 8: Figure S7A-C). Even though apoE2 correlated with t-tau and p-tau in all individuals (rho = 0.45–0.46, *p* < 0.05) (Additional file 8: Figure S7A) and in the Aβ− group (rho = 0.49–0.52, *p* < 0.05) (Additional file 8: Figure S7C), there was no such relationship in Aβ+ individuals (Additional file 8: Figure S7B).

### Follow-up data

In the longitudinal data, there was no significant difference in the CSF concentrations of total apoE or specific apoE isoforms (*p* > 0.05).

## Discussion

*APOE*-ε4 is the strongest risk factor of sporadic AD. The mechanisms underlying this association are, however, still unknown despite intense research for more than 20 years. Several processes potentially explaining the association of *APOE*-ε4 with AD have been suggested, including cholesterol transport, synaptic plasticity, Aβ clearance, and destabilization of microtubules [48], as well as direct toxicity from certain apoE fragments generated upon neuronal stress [49]. However, it is not fully understood if the relation of *APOE*-ε4 and AD is mediated by apoE directly or there are other mechanisms that drive apoE-associated risk for AD. In addition, data on the possible association of apoE concentrations in CSF and AD are inconsistent [22–29]. To our knowledge, this is the largest study so far examining the total apoE and the apoE isoform concentrations in human CSF, involving 1820 individuals. This study shows a significant increase in CSF total apoE concentration in Aβ+ compared with Aβ− groups (Fig. 1a, b). Even though the differences between the groups were statistically significant, the small effect size (AUC = 0.53–0.55) shows that the model has no discrimination capacity to distinguish between the groups. The large sample size triggers the differences to be statistically significant, while these effects are trivial and might lack clinical relevance. Moreover, the finding that there is no change in total apoE between clinically diagnosed AD and CU Aβ− group (Fig. 2a) suggests that CSF apoE concentrations do not explain the association of *APOE-*ε4 carrier status and increased risk of AD. Instead, the differential effect of *APOE-*ε4 on AD prevalence might be related rather to loss of function (apoE2), gain of toxic function (apoE4) [9, 10] or other mechanisms that are directly or indirectly mediated by apoE. In addition, CSF apoE concentrations could not be linked to cognitive status as determined by MMSE test scores (Additional file 8: Figure S7), which is in line with previous studies [24, 32, 50], indicating that CSF apoE concentrations are not informative for the diagnosis of AD.

There was no relationship between the concentration of total apoE and *APOE* genotype (Fig. 1e, f), which is in line with previous studies [24, 29].

The association between *APOE-*ε4 allele and PD is controversial [51–55], although *APOE-*ε2 allele increases the risk for sporadic PD [56]. The mechanisms underlying this association are unknown and the isoform-specific apoE concentrations in CSF can be hypothesized. To the best of our knowledge, this is the first study describing the effect of CSF apoE isoform concentrations in PD (Fig. 2). Here, PD was the only neurodegenerative disease with decreased CSF apoE2 concentrations compared to the CU Aβ− group (Fig. 2b). Further, the difference was not only statistically significant but also substantially altered. This observation is very interesting since *APOE*-ε2 is associated with higher prevalence of PD [56]. CSF apoE2 concentrations in PDD also showed a trend to be decreased when compared to CU Aβ− group (Fig. 2b), although this difference was not significant. Even though the CSF apoE3 concentrations were significantly higher in CU Aβ− individuals when compared to clinically diagnosed MCI, AD, PD, and PDD groups (Fig. 2c), these differences were minor (AUC = 0.55–0.61). Interestingly, CSF apoE4 concentration did not differ between CU Aβ− group and clinically diagnosed MCI, AD, PD, PDD, or DLB (Fig. 2d). There was no significant difference in CSF apoE concentrations between CU Aβ− and DLB patients, which is in contrast with previously published data [30] where CSF apoE protein levels were increased in DLB patients.

Not strong, but statistically significant correlations were found between AD biomarkers (Aβ_42/40_, t-tau, and p-tau) and CSF total apoE concentrations (Additional file 8: Figure S7), possibly due to the large sample size. The lack of correlation of CSF apoE2 concentrations and Aβ_42/40_ (Additional file 8: Figure S7) indicates that apoE isoforms might be differentially associated with Aβ pathology, where the apoE2 isoform is possibly not related to amyloidosis.

The increased concentration of apoE isoforms in heterozygous individuals in an isoform-dependent manner (E2 < E3 < E4) (Fig. 3) might be associated with the isoform-specific differences on Aβ clearance [9, 10], the mechanism being the least efficient for *APOE*-ε4. Previously, it was shown that in the mouse model of β-amyloidosis expressing human apoE isoforms, the clearance of Aβ in the brain interstitial fluid was the most efficient in homozygous APOE-ε2 carriers and the least in APOE-ε4 homozygotes, in both aged and young mice [57]. Similar findings were reported in bioengineered human vessels, where apoE4 is less effective than apoE2 in Aβ clearance [58]. These findings suggest that apoE isoforms contribute to AD risk by differentially regulating clearance of Aβ from the brain. The discrepancy in Aβ clearance among apoE isoforms might also be related to their differential ability to bind to lipoprotein receptors (E2 < E3 < E4) as well as to heparan sulfate proteoglycans (HSPGs), which were observed to promote tau pathology [59], Aβ aggregation, and microglial-mediated inflammatory response to amyloid [60, 61]. ApoE-4 showed the highest heparin-binding ability compared to other isoforms [62], and it is believed that weakened apoE-HSPG binding might have a therapeutic potential. *APOE* knockout leads to decreased aggregation of Aβ [62, 63], suggesting that apoE expression is critical for amyloid deposition. Moreover, *APOE-*ε4 carriers have increased amyloid plaque burden compared with non *APOE-*ε4 carrier AD patients [64]. Higher CSF apoE4 concentrations compared to other APOE isoforms in heterozygous individuals might be a partial explanation for why the *APOE*-ε4 genotype is associated with the accumulation of Aβ fibrils. However, the association of the concentration of apoE in CSF and Aβ aggregation cannot be inferred from this study and further research is needed. Similar shifts in the concentrations between the two isoforms in apoE from heterozygous individuals in CSF was previously observed by Martinez-Morillo et al. [24] and by Baker-Nigh et al. [26]. These changes were visible in both the Aβ+ and Aβ− groups, suggesting that CSF apoE concentration is not associated with AD pathology.

CSF apoE3 concentrations were significantly lower in *APOE-*ε3/ε4 patients compared to apoE3,3 and *APOE-*ε2/ε3 patients in all individuals as well as in both Aβ+ and Aβ− groups (Fig. 3). Unlike apoE3, the CSF concentration of apoE2 and apoE4 was similar between various genotypes (Fig. 3), most probably due to the lower number of individuals.

No change in total or isoform apoE concentrations were observed in longitudinal data, indicating that CSF apoE concentrations are unsuitable for monitoring the progression of AD.

The strengths of this study are the large cohort of well-characterized clinical samples, the presence of *APOE-*ε2/ε2 individuals in the cohort, and the access of apoE isoform data. The small sample consumption (20 μL of CSF) is an advantage of the method.

A limitations of this study are low number of clinically diagnosed AD-carrying *APOE*-ε2 allele and high analytical variability of the apoE4 peptide, which was therefore not used to measure the apoE4 concentrations in CSF. The high variability might be caused by lower thermal and chemical stability of the apoE4 peptide compared with other apoE isoforms [65]. To overcome this limitation, the peptides unique for E2/E3 and E3/E4 were used for calculation of isoform-specific apoE concentrations instead. The high correlation between the apoE2 peptide and the difference (total apoE − E3/E4 endogenous peptide) in *APOE-*ε2 carriers (rho = 0.935, *p* < 0.001) (Additional file 6: Figure S5A) supports the approach of using peptides unique for E2/E3 and E3/E4 to obtain isoform-specific apoE concentrations. In comparison, there was a moderate correlation between apoE4 peptide and the difference (total apoE − E2/E3 endogenous peptide) in *APOE-*ε4 carriers (rho = 0.750, *p* < 0.001) (Additional file 6: Figure S5B).

## Conclusion

In this large study, involving 1820 individuals, the total apoE and the apoE isoform concentrations in human CSF has been examined. In conclusion, total and isoform-specific apoE concentrations in CSF do not seem to be associated with AD diagnosis, cognitive impairment, or rate of decline.

## Supplementary information

Additional file 1: **Table S1.** The clinical diagnoses of the patients included in the study. **Table S2.** ApoE peptides used in the PRM-MS assay with acquisition characteristics. **Table S3.** The adjustment of internal standard (IS) concentrations.

Additional file 2: **Figure S1.** The LC gradient profile. Acquisition schematics (A) with the region of data collection expanded (B). Separation was performed at a flow rate of 300 μL/min with a broken gradient going from 0 to 30% B over 5.5 min. The set gradient is shown in pink, while the actual conditions at the time of spraying (the time delay due to the total delay volume of the LC system was about 3.15 min) are shown in blue. The 30 s peptide acquisition traces are shown in green (endogenous peptide) and orange (internal standard) with the peptide sequences indicated (* indicates peptides common to all isoforms). At most, six analytes were measured at the same time.

Additional file 3: **Figure S2.** Examples of chromatographic traces of the endogenous and internal standard (IS) peptides. The top part of each panel shows the chromatographic traces of the sum of the fragment ion peaks for the endogenous (red) and the IS (blue) peptides. The middle part shows the traces of the individual fragment ions for the endogenous peptide and the bottom part the traces of the individual fragment ions for the IS peptide. The peptides are LGADMEDVCGR (A), LGADMEDVR (B), CLAVYQAGAR (C), LAVYQAGAR (D), LGPLVEQGR (E) and LQAEAFQAR (F).

Additional file 4: **Figure S3.** The CSF Aβ_42_/ Aβ_40_ concentration ratio cut-off. The cut-off of Aβ_42_/ Aβ_40_ equal to 0.091 was determined by maximizing concordance and was used to dichotomize patients into amyloid β-positive (Aβ+) and amyloid β-negative (Aβ−) groups.

Additional file 5: **Figure S4.** Correlations between apoE peptides. The correlation between two peptides common in all three isoforms (LGPLVEQGR, LQAEAFQAR) (A). Total apoE concentrations correspond to the average of two common peptides (LGPLVEQGR, LQAEAFQAR). The correlations of total apoE with the peptides unique for E3/E4 (LAVYQAGAR) (B) and E2/E3 (LGADMEDVCGR) (C).

Additional file 6: **Figure S5.** Correlations between peptides. Correlation between E2 isoform specific peptide and the difference between total apoE and E3/E4 endogenous peptide in *APOE-*ε2 carriers (A) as well as between E4 isoform-specific peptide and the difference between total apoE and E2/E3 endogenous peptide in *APOE-*ε4 carriers (B). Both correlations were significant at the 0.01 level (2-tailed) with *p* < 0.001.

Additional file 7: **Figure S6.** Weighted linear fit reversed calibration curves. The graphs show the IS-to-endogenous peptide ratios plotted vs the amount of spiked IS in two different CSF pools: CSF pool 1 (A) and CSF pool 2 (B). The curve fits were obtained using weighted sum of squares (1/Y^2^). Both axes are logarithmic in order to separate the data points evenly.

Additional file 8: **Figure S7.** Correlation matrix for all individuals (A) and in amyloid β-positive (B) and β-negative (C) groups. Sig. indicates *p*-value, where: ** = Correlation is significant at the 0.01 level (2-tailed). * = Correlation is significant at the 0.05 level (2-tailed).

## Data Availability

The datasets used and analyzed during the current study are available from the corresponding author on reasonable request.

## Abbreviations

AD: Alzheimer’s disease
apoE: Apolipoprotein E
AUC: Area under the curve
Aβ: Amyloid β
CSF: Cerebrospinal fluid
CU: Cognitively unimpaired
DLB: Dementia with Lewy bodies
HSPGs: Heparin sulfate proteoglycans
IS: Internal standard
MCI: Mild cognitive impairment
MMSE: Mini-Mental State Examination
MSA: Multiple system atrophy
PD: Parkinson’s disease
PDD: Parkinson’s disease dementia
PSP: Progressive supranuclear palsy
ROC: Receiver operating characteristic
SCD: Subjective cognitive decline
