# Supplementary material for: Quantification of total apolipoprotein E and its isoforms in cerebrospinal fluid from patients with neurodegenerative diseases
Source: Alzheimers Res Ther. 2020 Feb 13;12:19. doi: 10.1186/s13195-020-00585-7 (PMC7020540; doi:10.1186/s13195-020-00585-7)
Supplement: Supplementary file 1 — Table S1. The clinical diagnoses of the patients included in the study. Table S2. ApoE peptides used in the PRM-MS assay with acquisition characteristics. Table S3. The adjustment of internal standard (IS) concentrations. [file 13195_2020_585_MOESM1_ESM.docx]

Supplementary table 1. The clinical diagnoses of the patients included in the study.

| Clinical diagnosis | N |
| --- | --- |
| Control* | 679 |
| Mild cognitive impairment | 309 |
| Alzheimer’s disease | 228 |
| Subjective cognitive decline* | 217 |
| Parkinson’s disease | 163 |
| Pervasive developmental disorder | 45 |
| Dementia with Lewy Body | 34 |
| Progressive supranuclear palsy | 28 |
| Multiple system atrophy | 26 |
| Vascular dementia | 13 |
| Frontotemporal dementia | 10 |
| Corticobasal syndrome | 7 |
| Corticobasal degeneration | 6 |
| Essential tremor | 4 |
| Semantic dementia | 5 |
| Normal pressure hydrocephalus | 2 |
| Progressive nonfluent aphasia | 2 |
| Creutzfeldt-Jakob disease | 1 |
| SNCA | 1 |
| Other atypical/unknown | 40 |

* Controls and subjective cognitive decline were combined together into cognitively unimpaired (CU) group (n=896)

Supplementary table 2. ApoE peptides used in the PRM-MS assay with acquisition characteristics.

| **Position**^a^ | **Sequence** | **Isoform** | **m/z**^b^ | **Charge, z** | **Fragment ions** | **NCE^c^** |
| --- | --- | --- | --- | --- | --- | --- |
| 104-114 | LGADMEDVCGR | apoE2/E3 | 611.76 | 2 | y3,y4,y5,y6,y7,y8,y9,y10 | 20 |
| 104-112 | LGADMEDVR | apoE4 | 503.24 | 2 | y3,y5,y6,y7,y8 | 20 |
| 158-167 | CLAVYQAGAR | apoE2 | 554.78 | 2 | y4,y5,y6,y7,y8,y9 | 20 |
| 159-167 | LAVYQAGAR | apoE3/E4 | 474.77 | 2 | y4,y5,y6,y7,y8 | 18 |
| 181-189 | LGPLVEQGR | common | 484.78 | 2 | y3,y4,y5,y6,y7 | 24 |
| 252-260 | LQAEAFQAR | common | 517.28 | 2 | y3,y4,y5,y6,y7,y8 | 18 |

^a^ – the position reflects to the amino acid sequences in the mature protein (without signal peptide)

^b^ – precursor m/z of the endogenous peptide

^c^ – normalized collision energy

Supplementary table 3. The adjustment of internal standard (IS) concentrations.

CSF concentrations for each of the apoE peptides were calculated by multiplying the endogenous-to-IS ratios of the summed fragment peak areas by the adjusted concentration of the corresponding IS. The adjustment of IS concentrations was performed by minimizing the sum of squared differences of the respective endogenous peptides’ concentrations using the Solver function in Microsoft Excel.

| **IS** | **Formula** |
| --- | --- |
| total apoE^a^ | $\sum_{all individuals} \left( LGPLVEQGR endogenous peptide-LQAEAFQAR endogenous peptide \right)^{2}$ |
| apoE3/E4^b^ | $\sum_{non-\varepsilon2 carriers} \left( total apoE - apoE3/E4 endogenous peptide \right)^{2}$ |
| apoE2/E3^c^ | $\sum_{non-\varepsilon4 carriers} \left( total apoE - apoeE2/E3 endogenous peptide \right)^{2}$ |

^a^ - the two peptides (LGPLVEQGR, LQAEAFQAR) common for all three isoforms were adjusted to their average and named total apoE

^b^ - the peptide unique for apoE3/E4 (LAVYQAGAR) was adjusted to total apoE

^c^ - the peptide unique for apoE2/E3 (LGADMEDVCGR) was adjusted to total apoE

The isoform quantification was not performed using apoE2 and apoE4 IS concentrations directly due to the high analytical variability of the apoE4 peptide. Instead, for concentration measurements in the heterozygote individuals, the difference between the common apoE and the respective isoform specific peptides were used as follows: for E3/4 heterozygotes: apoE4 = total apoE - apoE2/3 ; for E2/3 heterozygotes: apoE2 = total apoE - apoE3/4.

Supplementary fig. 1.

The LC gradient profile.

Acquisition schematics (A) with the region of data collection expanded (B). Separation was performed at a flow rate of 300 µL/min with a broken gradient going from 0 to 30% B over 5.5 min. The set gradient is shown in pink, while the actual conditions at the time of spraying (the time delay due to the total delay volume of the LC system was about 3.15 min) ae shown in blue. The 30 s peptide acquisition traces are shown in green (endogenous peptide) and orange (internal standard) with the peptide sequences indicated (* indicates peptides common to all isoforms). At most, six analytes were measured at the same time.

Supplementary fig. 2.

Examples of chromatographic traces of the endogenous and internal standard (IS) peptides.

The top part of each panel shows the chromatographic traces of the sum of the fragment ion peaks for the endogenous (red) and the IS (blue) peptides. The middle part shows the traces of the individual fragment ions for the endogenous peptide and the bottom part the traces of the individual fragment ions for the IS peptide. The peptides are LGADMEDVCGR (A), LGADMEDVR (B), CLAVYQAGAR (C), LAVYQAGAR (D), LGPLVEQGR (E) and LQAEAFQAR (F).

Supplementary fig. 3.

The CSF Aβ_42_/ Aβ_40_ concentration ratio cut-off.

The cut-off of Aβ_42_/ Aβ_40_ equal to 0.091 was determined by maximizing concordance and was used to dichotomize patients into amyloid β-positive (Aβ+) and amyloid β-negative (Aβ-) groups.

Supplementary fig. 4.

Correlations between apoE peptides.

The correlation between two peptides common in all three isoforms (LGPLVEQGR, LQAEAFQAR) (A). Total apoE concentrations correspond to the average of two common peptides (LGPLVEQGR, LQAEAFQAR). The correlations of total apoE with the peptides unique for E3/E4 (LAVYQAGAR) (B) and E2/E3 (LGADMEDVCGR) (C).

Supplementary fig. 5.

Correlations between peptides.

Correlation between E2 isoform specific peptide and the difference between total apoE and E3/E4 endogenous peptide in *APOE-*ε2 carriers (A) as well as between E4 isoform-specific peptide and the difference between total apoE and E2/E3 endogenous peptide in *APOE-*ε4 carriers (B).

Both correlations were significant at the 0.01 level (2-tailed) with p<0.001.

Supplementary fig. 6

Weighted linear fit reversed calibration curves. The graphs show the IS-to-endogenous peptise ratios plotted vs the amount of spiked IS in two different CSF pools: CSF pool 1 (A) and CSF pool 2 (B). The curve fits were obtained using weighted sum of squares (1/Y^2^). Both axes are logarithmic in order to separate the data points evenly.

Supplementary fig. 7

Correlation matrix for all individuals (A) and in amyloid β-positive (B) and β-negative (C) groups.

Sig. indicates p-value, where:

** = Correlation is significant at the 0.01 level (2-tailed).

* = Correlation is significant at the 0.05 level (2-tailed).
