# Supplementary figures and images for: Quantification of total apolipoprotein E and its isoforms in cerebrospinal fluid from patients with neurodegenerative diseases
Source: Alzheimers Res Ther. 2020 Feb 13;12:19. doi: 10.1186/s13195-020-00585-7 (PMC7020540; doi:10.1186/s13195-020-00585-7)

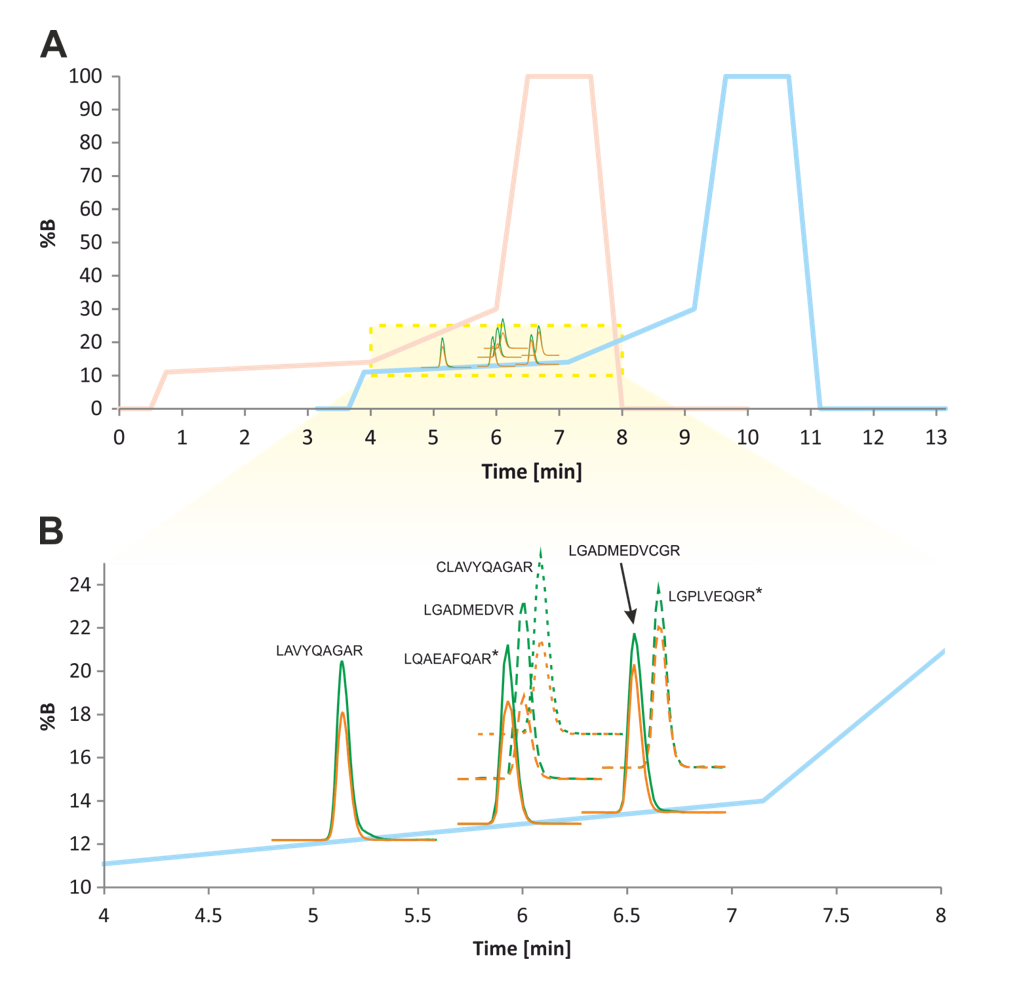

Supplement: Supplementary file 2 — Figure S1. The LC gradient profile. Acquisition schematics (A) with the region of data collection expanded (B). Separation was performed at a flow rate of 300 μL/min with a broken gradient going from 0 to 30% B over 5.5 min. The set gradient is shown in pink, while the actual conditions at the time of spraying (the time delay due to the total delay volume of the LC system was about 3.15 min) are shown in blue. The 30 s peptide acquisition traces are shown in green (endogenous peptide) and orange (internal standard) with the peptide sequences indicated (* indicates peptides common to all isoforms). At most, six analytes were measured at the same time. [file 13195_2020_585_MOESM2_ESM.tif]

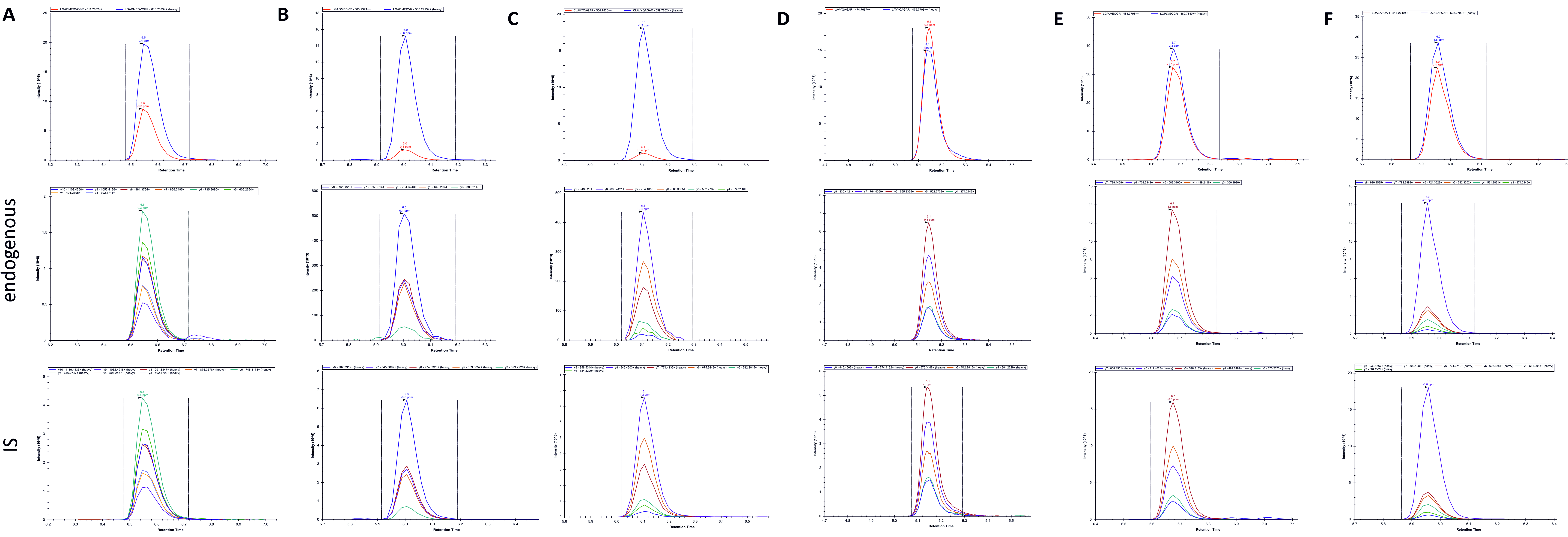

Supplement: Supplementary file 3 — Figure S2. Examples of chromatographic traces of the endogenous and internal standard (IS) peptides. The top part of each panel shows the chromatographic traces of the sum of the fragment ion peaks for the endogenous (red) and the IS (blue) peptides. The middle part shows the traces of the individual fragment ions for the endogenous peptide and the bottom part the traces of the individual fragment ions for the IS peptide. The peptides are LGADMEDVCGR (A), LGADMEDVR (B), CLAVYQAGAR (C), LAVYQAGAR (D), LGPLVEQGR (E) and LQAEAFQAR (F). [file 13195_2020_585_MOESM3_ESM.tif]

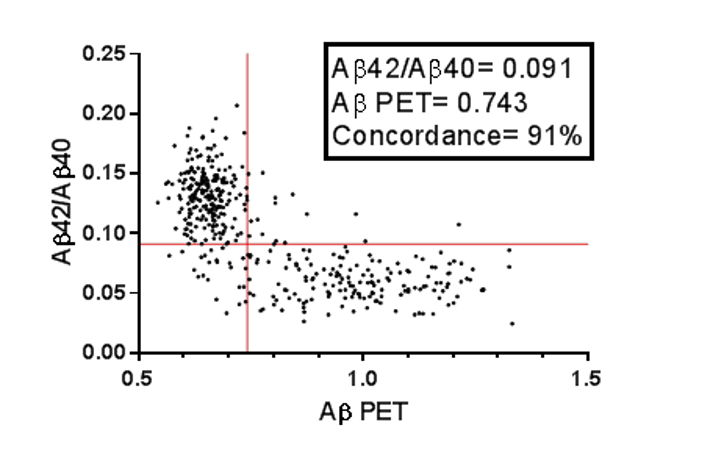

Supplement: Supplementary file 4 — Figure S3. The CSF Aβ42/ Aβ40 concentration ratio cut-off. The cut-off of Aβ42/ Aβ40 equal to 0.091 was determined by maximizing concordance and was used to dichotomize patients into amyloid β-positive (Aβ+) and amyloid β-negative (Aβ−) groups. [file 13195_2020_585_MOESM4_ESM.tif]

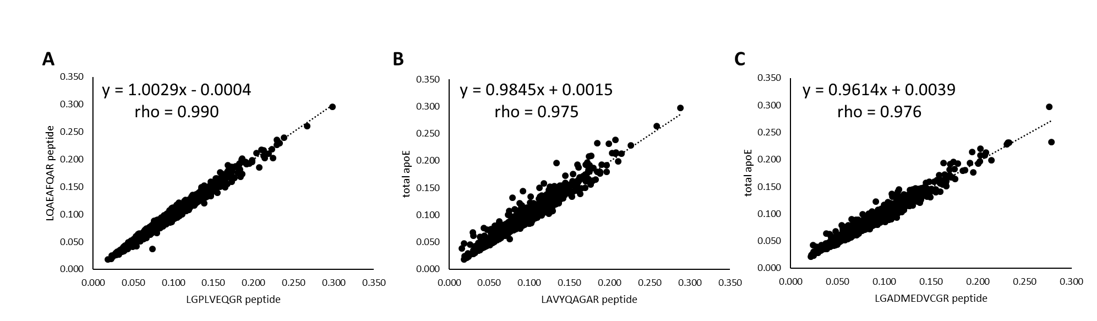

Supplement: Supplementary file 5 — Figure S4. Correlations between apoE peptides. The correlation between two peptides common in all three isoforms (LGPLVEQGR, LQAEAFQAR) (A). Total apoE concentrations correspond to the average of two common peptides (LGPLVEQGR, LQAEAFQAR). The correlations of total apoE with the peptides unique for E3/E4 (LAVYQAGAR) (B) and E2/E3 (LGADMEDVCGR) (C). [file 13195_2020_585_MOESM5_ESM.tif]

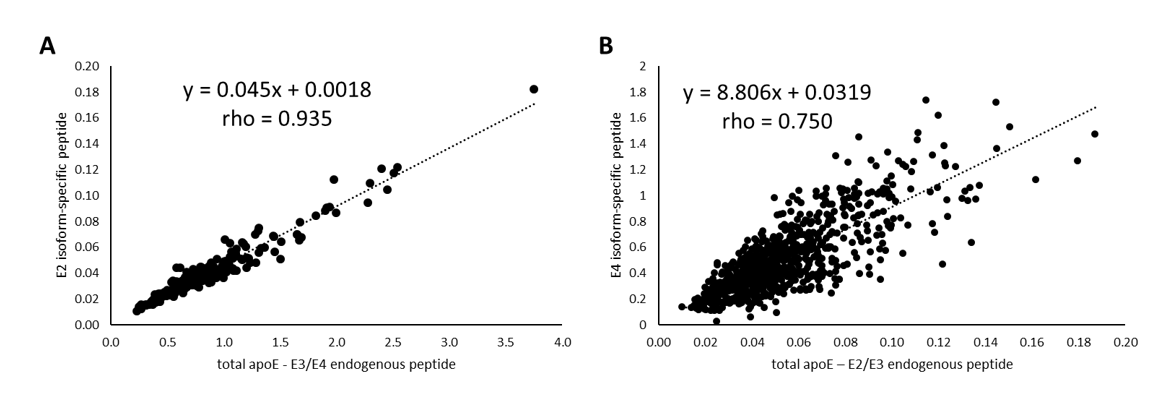

Supplement: Supplementary file 6 — Figure S5. Correlations between peptides. Correlation between E2 isoform specific peptide and the difference between total apoE and E3/E4 endogenous peptide in APOE-ε2 carriers (A) as well as between E4 isoform-specific peptide and the difference between total apoE and E2/E3 endogenous peptide in APOE-ε4 carriers (B). Both correlations were significant at the 0.01 level (2-tailed) with p < 0.001. [file 13195_2020_585_MOESM6_ESM.tif]

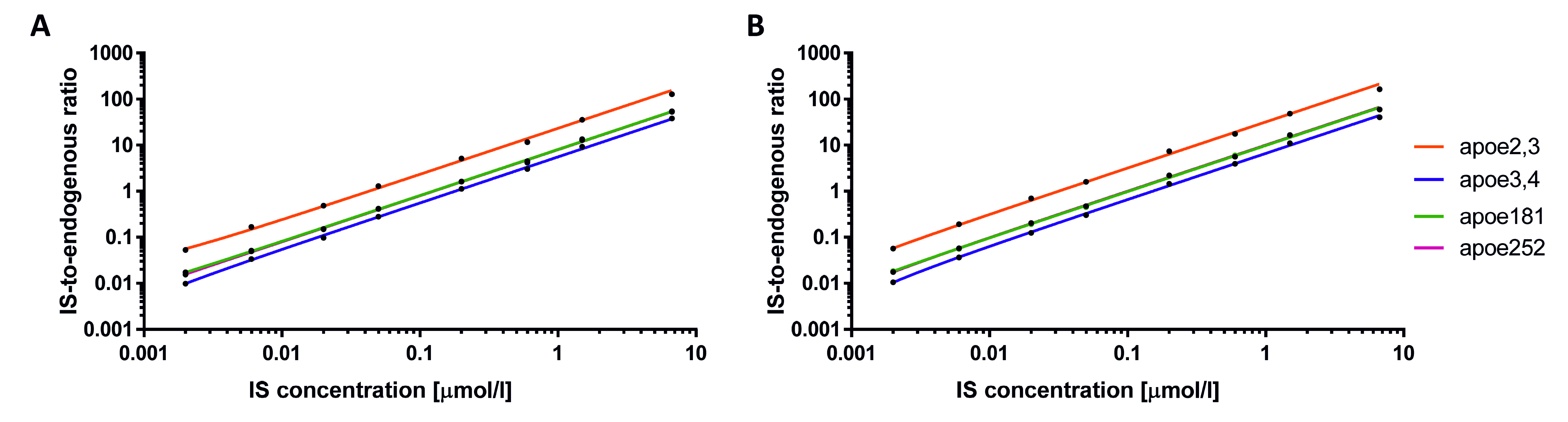

Supplement: Supplementary file 7 — Figure S6. Weighted linear fit reversed calibration curves. The graphs show the IS-to-endogenous peptide ratios plotted vs the amount of spiked IS in two different CSF pools: CSF pool 1 (A) and CSF pool 2 (B). The curve fits were obtained using weighted sum of squares (1/Y2). Both axes are logarithmic in order to separate the data points evenly. [file 13195_2020_585_MOESM7_ESM.tif]

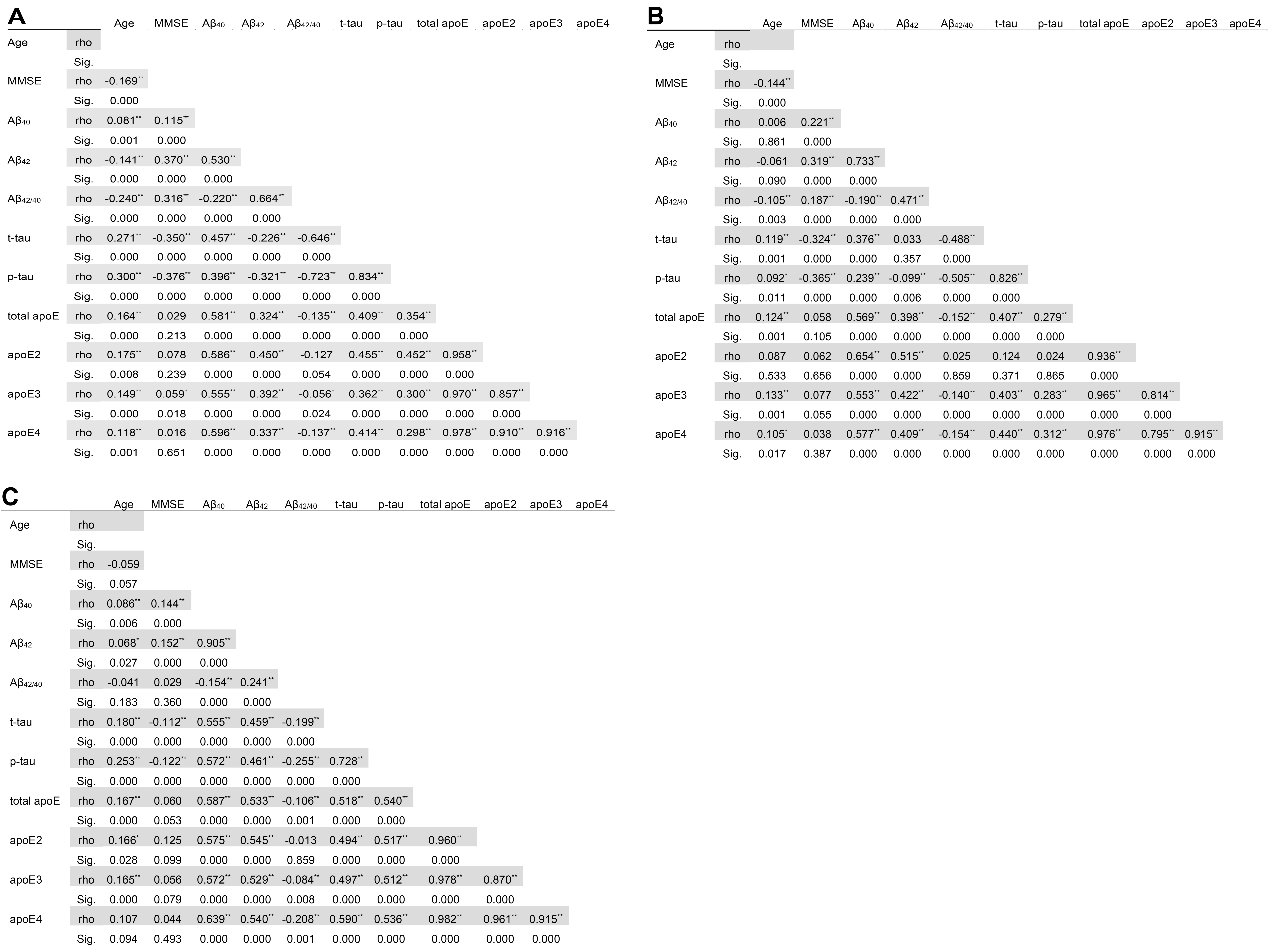

Supplement: Supplementary file 8 — Figure S7. Correlation matrix for all individuals (A) and in amyloid β-positive (B) and β-negative (C) groups. Sig. indicates p-value, where: ** = Correlation is significant at the 0.01 level (2-tailed). * = Correlation is significant at the 0.05 level (2-tailed). [file 13195_2020_585_MOESM8_ESM.tif]
